# Development and Validation of a Hydrophilic Interaction Liquid Chromatography Tandem Mass Spectrometry Method for the Determination of Asparagine in Human Serum

**DOI:** 10.1155/2020/6980392

**Published:** 2020-02-28

**Authors:** Haoyang Lu, Xiaoyun Zeng, Lihua Yu, Zhanzhang Wang, Danna Lin, Xiaojia Ni, Dewei Shang, Ming Zhang, Jinqing Hu, Shuhua Deng, Xiuqing Zhu, Yuqing Chen, Huanshan Xie, Lihua Yang, Yuguan Wen

**Affiliations:** ^1^The Affiliated Brain Hospital of Guangzhou Medical University (Guangzhou Huiai Hospital), No. 36, Mingxin Road, Guangzhou 510370, China; ^2^Seventh Affiliated Hospital of Sun Yat-Sen University, Shenzhen 518107, China; ^3^Zhujiang Hospital of Southern Medical University, No. 253, Industrial Avenue, Guangzhou 501280, China

## Abstract

L-Asparagine (ASN) is the catalyze substrate of L-asparaginase (ASNase), which is an important drug for acute lymphoblastic leukemia (ALL) patients. The ASN level is found to be closely associated with the effectiveness of ASNase treatment. In this study, a hydrophilic interaction liquid chromatography tandem mass spectrometry (HILIC-MS/MS) method was developed for the determination of ASN in the human serum using a stable isotope-labeled internal standard (ASN-D_3_). Serum samples were prepared by a one-step precipitation procedure using methanol and separated by an Agilent HILIC Plus column with the mobile phase of methanol-water (95 : 5, v/v, containing 5 mM ammonium formate and 0.1% formic acid), at a constant flow rate of 0.3 mL/min. Mass spectrometric analysis was conducted using multiple-reaction monitoring in the positive electrospray ionization mode. Serum ASN concentrations were determined over a linear calibration curve range of 2–200 *μ*M, with acceptable accuracies and precisions. The validated HILIC-MS/MS method was successfully applied to the quantification of ASN levels in the serum from patients with ALL. Collectively, the research may shed new light on an alternative rapid, simple, and convenient quantitative method for determination of serum ASN in ALL patients treated with ASNase.

## 1. Introduction

Acute lymphoblastic leukemia (ALL) is the most common cancer among children and is characterized by overproduction and accumulation of immature lymphoid cells [1, 2, 3]. The cure rate of ALL has exceeded 80% due to the development of therapeutic approaches, of which L-asparaginase (ASNase) played a prominent role [4, 5]. ASNase exerts its antileukemic activity by catalyzing the hydrolysis of L-asparagine (ASN), which is a nonessential amino acid that normal cells are able to synthesize, while leukemic cells have to uptake from the intercellular fluid. The depletion of ASN leads to inhibition of protein and RNA synthesis in leukemic cells, eventually inducing apoptosis [4, 6]. Reports have shown that the normal ASN level in the human serum is 40–80 *μ*M and ASN depletion of over 90% or a serum ASN level below 3 *μ*M is considered to be effective for the treatment of ALL [7, 8, 9]. Although the incidence of adverse events such as hypersensitivity reactions has been reduced significantly since the polyethylene glycol- (PEG-) conjugated ASNase was used clinically, drug resistance of ASNase continues to provide challenges for continuing ASNase treatment [10, 11, 12].

Much progress has been made in understanding the mechanisms involved in the drug resistance of ASNase. Besides high expression and activity of ASN, host factors, especially ASNase antibody production, were also reported to be associated with the drug resistance of ASNase. However, it has been reported that the host factors would eventually result in high expression and activity of ASN [10, 13, 14]. Therefore, to guide the decision of switching preparations or dose escalation of ASNase, the monitoring of drug concentration or therapeutic markers were necessary for ALL patients. The ASNase level, ASNase antibody titer, and ASN level are all available for monitoring during ASNase treatment. Of these, measurement of the ASN level is the most ideal means for applying due to its superiority in reflecting the final outcome of all variables including dietary supplementation of the amino acid [15].

Reversed-phase high-performance liquid chromatography (RP-HPLC) with fluorescence detection and liquid chromatography tandem mass spectrometry (LC-MS/MS) are two alternative methods with high sensitivity and selectivity to be used for quantitative analysis of ASN concentrations in biological samples including plasma, serum, and cerebrospinal fluid [16, 17, 18]. However, the widespread use of RP-HPLC with fluorescence for measuring the ASN level is hampered by the complexity process since ASN has to be precolumn derivatized with *o*-phthaldialdehyde, while the use of LC-MS/MS would encounter the hurdle of the high matrix effect because the strongly polar ASN is difficult to separate from endogenous components through the commonly used RP-HPLC column. Therefore, there is an urgent demand for a rapid, simple, and convenient method for ASN determination in ALL patients treated with ASNase. Hydrophilic interaction liquid chromatography (HILIC) is an alternative HPLC method based on the strong hydrophilic interaction of the hydrophilic polar stationary phase with highly polar compounds such as amino acids. HILIC could overcome the drawback of poor solubility, poor analytical reproducibility, and limited compatibility with electrospray ionization mass spectrometry that are often encountered in normal-phase HPLC. It was observed that analytes can be eluted similarly to normal phase liquid chromatography in the mobile phase of a reversed-phase system [19, 20, 21]. Therefore, HILIC may hold promise as an alternative method for quantitative analysis of ASN concentrations.

In the present study, a rapid, simple, and convenient method was developed for determination of ASN in the human serum using one-step sample protein precipitation and analysis by HILIC-MS/MS. A complete validation was conducted, including assessment of linearity, lower limit of quantification (LLOQ), specificity, carryover, matrix effect, recovery, accuracy, precision, and stability. Furthermore, this method was successfully applied to the determination of ASN in the serum of ALL patients and was effectively used to guide the rational clinical use of ASNase. The method may have potential application for serum ASN determination in ALL patients treated with ASNase.

## 2. Materials and Methods

### 2.1. Standards and Chemicals

L-Asparagine monohydrate (chemical purity 98%) and L-asparagine-D_3_ (ASN-D_3_, isotope-labeled internal standard of asparagine, chemical purity 98%, isotopic purity 98.3%) were purchased from Toronto Research Chemicals Inc. (Toronto, Canada). Ammonium formate (MS grade) and activated charcoal were supplied by Sigma-Aldrich Co., LLC (St. Louis, MO, USA). Formic acid (HPLC grade) was provided by Dikma Technologies Inc. (Lake Forest, CA, USA). Pegaspargase injection (5 mL: 3750 IU) was purchased from Hengrui Medicine (Jiangsu, China). Methanol (HPLC grade) was obtained from Merck KGaA (Darmstadt, Germany). Purified water (resistivity 18 MΩ) was prepared using a Milli-Q water purification system (Millipore Corporation, Billerica, MA, USA). Human serum was obtained from consenting healthy volunteers at the Affiliated Brain Hospital of Guangzhou Medical University.

### 2.2. Instrument and Conditions

Chromatographic analysis was performed using a Shimadzu 20A HPLC system consisting of two LC-20AD pumps, a DGU-20A_3R_ degassing unit, an SIL-20A autosampler, and a CTO-20A column oven (Shimadzu Corporation, Kyoto, Japan). Separations were conducted at 40°C on an HILIC Plus analytical column (4.6 × 100 mm, particle size 3.5 *μ*m, Agilent Technologies Inc., Santa Clara, CA, USA) with a mobile phase of methanol-water (95 : 5, v/v, containing 5 mM ammonium formate and 0.1% formic acid) at a flow rate of 0.3 mL/min. The total run time was 6 min, and the injection volume was 5 *μ*L.

**Figure 1:**
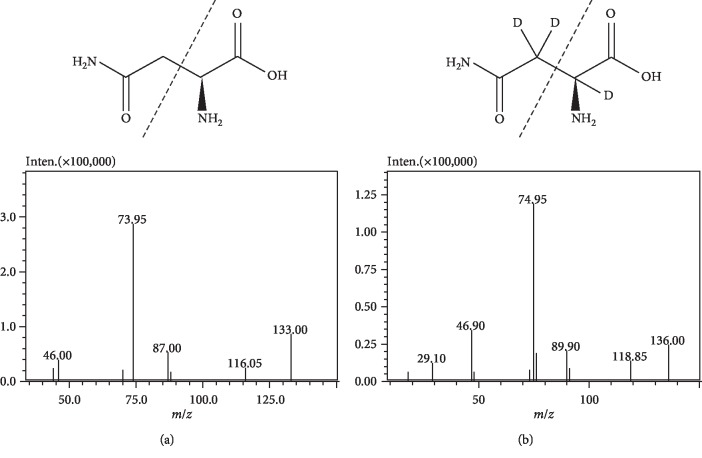
Molecular structure and product ion spectra of ASN (a) and ASN-D_3_ (b).

MS detection was carried out using a Shimadzu LCMS-8040 triple-quad mass spectrometer (Shimadzu Corporation) equipped with an electrospray ionization source operating in the positive mode (ESI^+^). Quantification was performed using the multiple-reaction monitoring mode. The interface voltage and conversion dynode voltage were 4.5 kV and 6 kV, respectively. DL temperature and heat block temperature were 250°C and 400°C, respectively. Nitrogen was used as the nebulizing and drying gas at flow rates of 3 and 15 L/min, respectively, while argon was employed as the collision gas at a pressure of 230 kPa. The ion transitions were *m/z* 133.00 ⟶ 73.95 and *m/z* 136.00 ⟶ 74.95 for ASN and ASN-D_3_, respectively (Figure 1).

Shimadzu LabSolutions Workstation (edition 1.0.5036.31919, Shimadzu Corporation) was used for data acquisition and processing, and Microsoft Office Excel 2007 (Redmond, WA, USA) was used to calculate means, standard deviations (SDs), relative standard deviations (RSDs), and coefficients of variation (CVs).

### 2.3. Preparation of ASN-Free Serum

**Figure 2:**
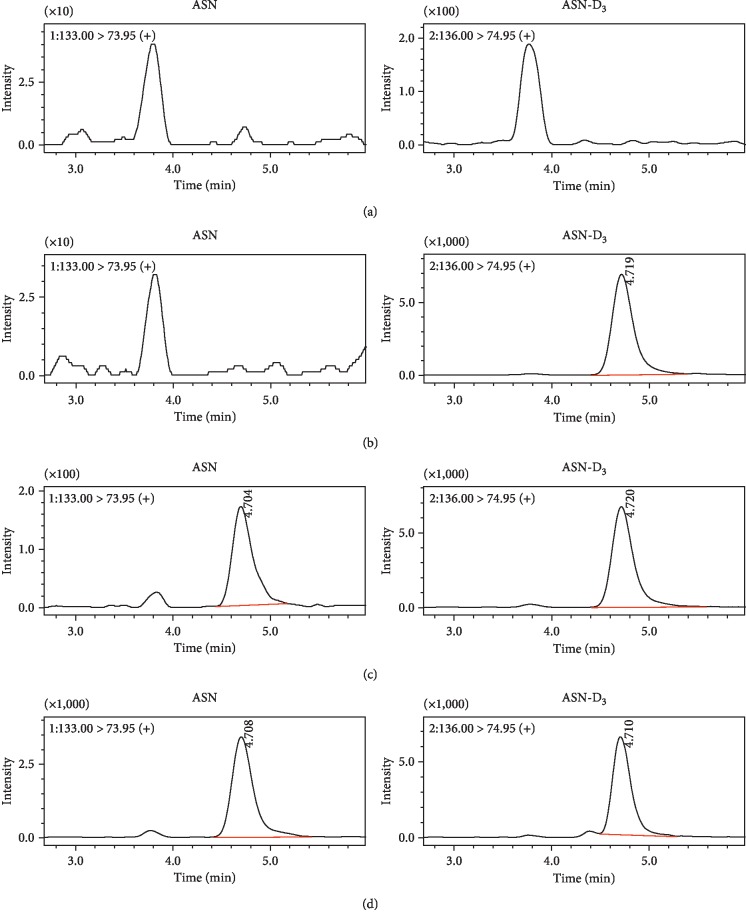
Typical chromatograms for the ASN-free human serum (a), ASN-free human serum spiked with 100 *μ*M of IS (b), ASN-free human serum spiked with 2 *μ*M of ASN (LLOQ) and 100 *μ*M of IS (c), and real human serum from ALL patients (d).

ASN-free serum was prepared by charcoal stripping [22, 23]. In brief, activated charcoal was added to the human serum at a ratio of 0.2 g/mL and stored at 4°C for 6 h. The mixture was then centrifuged at 20,000*g* for 10 min. The supernatant was then stripped by repeating the above process twice. The stripped human serum was confirmed to be free of ASN by HILIC-MS/MS (Figure 2(a)).

### 2.4. Standard Solutions

Stock solutions of ASN (100 mM) were prepared in water and stored at −20°C. Standard solutions for the calibration curve were prepared by serial dilution of the ASN stock solution with 50% methanol. The standard solutions were used to spike the ASN-free (charcoal-stripped) human serum to provide mimic serum samples containing ASN at final concentrations of 2, 4, 20, 40, 100, 160, and 200 *μ*M. Quality control (QC) samples were prepared in the same way at ASN concentrations of 6, 80, and 150 *μ*M.

### 2.5. Serum Sample Preparation

ASN was extracted from the serum by protein precipitation. In brief, blood samples were collected into a blood collection tube containing the coagulant. After shaking gently, blood samples were placed at 4°C for 5 min and then centrifuged at 4°C and 3000*g* for 5 min. The supernatant, which was serum, was transferred to fresh tubes. Then, serum samples (100 *μ*L) were immediately deproteinized by addition of 500 *μ*L methanol and stored at −70°C until analysis. For the determination of ASN, 20 *μ*L IS (500 *μ*M ASN-D_3_) and 10 *μ*L formic acid (5%, v/v) were added to the deproteinized samples and vortexed for 1 min. The mixtures were then centrifuged at 20,000*g* for 5 min. Supernatants were collected for HILIC-MS/MS analysis.

### 2.6. Method Validation

The HILIC-MS/MS method was validated for the determination of ASN according to the recommendations issued by the Pharmacopoeia of the People's Republic of China (Edition 2015) and in compliance with the principles of guidance of the US FDA and European Medicines Agency. A complete validation, including assessment of linearity, LLOQ, selectivity, carryover, matrix effect, recovery, accuracy, precision, and stability, was carried out.

#### 2.6.1. Specificity, Sensitivity, and Linearity

Selectivity for ASN and isotope-labeled IS (ASN-D_3_) relative to the endogenous matrix was tested in the ASN-free (charcoal-stripped) human serum. Blank samples were prepared and analyzed as described in Section 2.3. Interfering substances at the retention time of ASN should be undetectable or less than 20% and 5% of the peak areas for the LLOQ (2 *μ*M) of the analyte and IS, respectively. The sensitivity was defined by the LLOQ of ASN, with acceptable precision (RSD ≤ 20%), accuracy (within ±20%), and signal-to-noise ratio (*S/N* ≥ 10). The calibration curve was designed according to the normal range of ASN concentrations in the human serum (40–80 *μ*M). The linearity range for ASN was 2–200 *μ*M. Peak area ratios of ASN and IS against the concentration ratios at 2, 4, 20, 40, 100, 160, and 200 *μ*M were plotted to establish the calibration curve using the least-squares regression equation, with a weighting factor of 1/*x*^2^.

#### 2.6.2. Precision and Accuracy

The intra- and interbatch precision and accuracy for ASN were investigated on three different occasions by analysis of spiked charcoal-stripped serum samples at the LLOQ (2 *μ*M) and three QC levels (6, 80, and 150 *μ*M) in six replicates. The CV was used to evaluate intra- and interbatch precision, with acceptance criteria ≤15% for low, medium, and high QC (LQC, MQC, and HQC) samples and ≤20% at the LLOQ concentration. The accuracy values should be 85%–115% of the nominal concentrations, except for the LLOQ, which was extended to 80%–120% of the nominal value.

#### 2.6.3. Matrix Effect and Recovery

The effect of the endogenous matrix in the serum on the ionization of ASN or IS was evaluated by comparing the relative responses of postspiked samples (analytes and IS solutions were added to postextracted ASN-free (charcoal-stripped) biological matrix residues) and unextracted samples (analytes and IS solutions were directly diluted using 50% methanol) at three QC levels. The matrix effect was expressed as the matrix factor normalized to IS. The evaluation of the matrix effect in the serum was processed in four replicates. The recovery rates of ASN and IS were measured by comparing the peak areas of the extracted samples, which were prepared as described in Section 2.5, with those of the unextracted samples at three QC levels with six replicates.

#### 2.6.4. Carryover

The carryover effect was investigated by injecting a blank sample (charcoal-stripped human serum) following an upper limit of quantification (ULOQ, 200 *μ*M) sample. The evaluation of carryover was processed in three replicates, and the peak areas of blank samples should not exceed 20% of the LLOQ and 5% of IS.

#### 2.6.5. Stability

Mimic charcoal-stripped human serum samples at the LQC and HQC, which were deproteinized by methanol, were analyzed together with freshly prepared calibration samples to obtain actual concentrations. The mean concentrations should be 85%–115% of the nominal concentrations to prove stability over different storage periods.

Stability of the stock solution was evaluated by comparing the response of an ASN solution stored at −20°C for 92 days with that of a freshly prepared solution. The physical and chemical properties of IS (ASN-D_3_) were considered to be similar to those of ASN, so the stock solution stability of IS was not assessed in this study. Benchtop stability of ASN was evaluated by storing QC samples prepared with the charcoal-stripped human serum at room temperature for 48 h prior to preparation. Long-term stability was assessed by storing QC samples at −70°C for 87 days. To evaluate whether reinjection or delayed injection of prepared samples would lead to degradation, processed QC samples were stored in the autosampler (room temperature) or refrigerator (4°C) for 48 h. To confirm whether the presence of ASNase in the human serum confounds the determination of ASN, ASNase was added to LQC and HQC charcoal-stripped human serum samples to a final activity of 2 IU/mL (C_max_ of ASNase under the therapeutic dose was 1-2 IU/mL). The mixtures were then immediately deproteinized by methanol and stored at room temperature for 48 h. The actual concentrations of these QC samples were determined using a freshly prepared calibration curve, and mean concentrations that were 85%–115% of the nominal concentrations at each level were considered to demonstrate stability.

### 2.7. Serum ASN Concentrations in ALL Patients

The validated HILIC-MS/MS method was used to monitor serum ASN concentrations in ALL patients. Serum samples (85) from consenting ALL patients at the Zhujiang Hospital of Southern Medical University were analyzed. Forty-nine of the 85 samples were from patients receiving ASNase therapy. Prior to analysis, an informed consent form was signed by each patient giving the right to use their serum sample for the research purpose.

## 3. Results and Discussion

### 3.1. LC-MS/MS Method Development

Several C18 and C8 columns were initially investigated for RP-HPLC during the optimization process. Regardless of whether ammonium formate or formic acid was added, acetonitrile offered a low response and poor peak shape for ASN using both C8 and C18 columns. Noticeably, the response and peak shape for ASN were greatly improved in the methanol-water system. However, in the methanol-water system, ASN was eluted quickly irrespective of pH, resulting in a significant matrix effect and that the retention time was barely changed and even the proportion of methanol was varied between 60% and 95% (data not shown). In conclusion, commonly used RP-HPLC was not available for determination of ASN concentration because of its high polarity.

To overcome these drawbacks, HILIC, an alternative method for the separation of polar compounds such as amino acids, was used. High polar compounds not only would have better retention and separation in the HILIC method but also do not require the traditional derivatization steps or ion-pairing separations [21]. The liquid chromatography was optimized on an HILIC column. An Agilent HILIC Plus column (4.6 × 100 mm, particle size 3.5 *μ*m) was employed with a mobile phase of methanol-water. At the initial stage of development, ammonium formate (5 mM) was added to the mobile phase. However, it was observed that the peak shape was broad with a little trailing, which was probably because ASN is an amphoteric compound. Both molecular and ionic forms of ASN, which have different polarities, were found to be present in the mobile phase. Therefore, 0.1% formic acid (v/v) was added to the mobile phase to transform all ASN into the ionic form. In the presence of acid, the peak shape was improved and the signal was enhanced. In addition, the flow rate of the mobile phase is another factor affecting spray efficiency and impacts the signal response in MS. Although a flow rate of 0.2 mL/min is recommended for the MS detector used in this study, a low flow rate usually leads to broad peak shapes and long analysis times (10 min for 0.2 mL/min in this research). Flow rates of 0.2–0.5 mL/min were evaluated, and, as expected, the signal response was higher at a low flow rate. However, at 0.3 mL/min, the ASN peak area was similar to that at 0.2 mL/min and the analysis time was shortened to 6 min. In consequence, 0.3 mL/min was the optimal flow rate of the mobile phase for determination of ASN.

### 3.2. Extraction Method Development

Traditional precipitation (PPT), liquid-liquid extraction (LLE), and solid-phase extraction (SPE) are three commonly used effective extraction methods of biological samples [24, 25, 26]. Of these, LLE is inappropriate for this study because ASN is highly water soluble and is difficult to extract using organic solvents such as ethyl acetate or chloroform. SPE has been favored for analysis of amino acids in the biological matrix because of its efficient enrichment of analytes and clearance of matrix components. However, preparation of biological samples using SPE is always costly and time-consuming. The current study aimed to develop a simple and rapid method which could be applied to serum ASN determination of ALL patients treated with ASNase. PPT was a quick and convenient extraction method and thus employed for the pretreatment of serum samples. Methanol and acetonitrile were evaluated as precipitation agents, and the baselines of samples treated with both agents were similar. It was observed that, in both cases, the peaks were branched and trailing, probably because of differences in pH between the sample media and the mobile phase. To adjust the pH of the sample media, 10 *μ*L 5% formic acid was added after the addition of IS. In the presence of formic acid, the peaks were smooth and symmetrical, and the signal response after methanol precipitation was higher. Therefore, methanol was selected as the precipitation agent in the current method.

Since ASNase is present in the serum of ALL patients, ASN in serum samples would be degraded during storage and preparation. In previous research, sulfosalicylic acid was reported to be capable of inactivating ASNase by nonspecific precipitation and thus widely used to precipitate the serum proteins after the serum sample was separated from whole blood [18]. Nevertheless, sulfosalicylic acid strongly impacted the peak shapes of ASN in HILIC, probably due to the change of pH. Methanol, another commonly used precipitation agent, is able to provide a similar function as sulfosalicylic acid. Therefore, in this research, methanol could play a dual role by inactivating ASNase and preparing the serum sample, and based on this, 500 *μ*L methanol was added to 100 *μ*L serum sample immediately after separation from whole blood. After methanol addition, the sample could be stored at −70°C or be further processed. It was shown that methanol provided a similar peak shape to ASN water solution. Under such circumstances, the LLOQ of ASN was 2 *μ*M, which is lower than the concentration (3 *μ*M) considered to be effective for the treatment of ALL. Besides, accuracy and precision were 99.44 ± 13.52% and 15.02% at the LLOQ, respectively. In summary, the rapid and convenient method that has been developed in this research, with a low LLOQ, is applicable to serum ASN quantification in ALL patients.

### 3.3. Method Validation

#### 3.3.1. Specificity, Sensitivity, Linearity, and Carryover

The specificity of the assay method was assessed by evaluating potential interference or background noise at the LC retention times (*t*_*R*_) for analytes and IS from endogenous compounds. The chromatograms of the ASN-free human serum, spiked charcoal-stripped serum, and normal serum from ALL patients were compared (Figure 2). The figure shows that *t*_*R*_ of ASN and IS was both approximately 4.7 min and that no chromatographic peak attributable to the matrix component was observed at the retention time of the analytes. Sensitivity was satisfactory in the determination of ASN, with signal-to-noise ratios (*S*/*N*) over 10.

Good linearity was observed over the range of 2–200 *μ*M, with correlation coefficients (*R*^2^) greater than 0.99 for all routine calibration curves, which was applicable to serum ASN determination in ALL patients. The slope and intercept were 2.2631 ± 0.7621 and −0.0095 ± 0.0083, respectively, and the calibration accuracy of all seven calibrated levels was less than ±15% bias. No carryover was observed in the charcoal-stripped samples after injections of ULOQ (200 *μ*M).

#### 3.3.2. Matrix Effects and Recovery

The matrix effect was assessed to evaluate the possible enhancement or suppression of ionization caused by endogenous components in the biological matrices. The peak area ratios of ASN and IS in postextracted samples and solvent samples were compared at equivalent concentrations. The IS-normalized matrix effects at three QC levels are summarized in Table 1. The mean IS-normalized matrix effects were 94.64%–100.91% and the CVs were less than 15%, indicating that the endogenous matrix did not affect the determination of ASN under the validated conditions.

The overall process recoveries (also known as absolute recoveries) of ASN at 6, 80, and 150 *μ*M were 60.32 ± 5.10%, 61.37 ± 0.91%, and 63.17 ± 1.57%, respectively, while the recovery of IS was 63.83 ± 2.12%. Collectively, the overall process recoveries of the method were acceptable.

#### 3.3.3. Precision and Accuracy

Six replicates of charcoal-stripped serum samples at the LLOQ and three QC levels were used to evaluate the precision and accuracy for ASN. The intra- and interbatch precisions were 5.03%–15.02% and 3.89%–14.74%, respectively, and the deviations from nominal values were 0.07%–8.20% (Table 2). Both precision and accuracy values satisfied the criteria in the guidelines, demonstrating that the validated method was reliable and reproducible for the determination of ASN.

#### 3.3.4. Stability

The recovery of ASN from the stock solution was 102.70% ± 4.08% after storage at −20°C for 92 days. The stability of ASN in precipitated charcoal-stripped serum samples is summarized in Table 3. After precipitation with methanol, ASN was stable for at least 48 h during benchtop preparation and after 87 days of storage at −70°C. Prepared samples also exhibited good stability after storage in the autosampler (room temperature) or when refrigerated at 4°C for 48 h. With the presence of high levels of ASNase in the charcoal-stripped human serum, no significant degradation was found at room temperature for 48 h with the addition of methanol, indicating that methanol could inhibit the activity of ASNase. Methanol-precipitated human serum samples can thus be accurately analyzed under laboratory conditions without significant degradation of ASN.

### 3.4. Method Application

By employing the developed HILIC-MS/MS method, we determined the concentrations of ASN in the serum sample collected from 49 ALL patients. Before administration of ASNase, the serum ASN concentration was 52.06 ± 13.11 *μ*M (36 samples, range 36.16–90.32 *μ*M). After ASNase therapy, ASN concentrations in 46 of the 49 serum samples were below the detection limit of this method, suggesting that the therapies for these 46 patients were effective. Residual ASN was still detected in the sera of three patients treated with ASNase (42.63, 17.60, and 23.60 *μ*M), indicating that the curative effects of ASNase in these three patients was unsatisfactory and that the clinical treatment should be optimized (Table 4). It was found that the ASN level was in correlation with the effectiveness of ASNase treatment. To meet satisfactory effects in the treatment of ALL, a serum ASN level should be below 3 *μ*M and ASNase should be higher than 0.1 IU/mL [27]. However, the ASNase activity of three patients with residual ASN (Nos. 21, 30, and 31) was found to be 0.017 IU/mL, 0.082 IU/mL, and 0.015 IU/mL, respectively, which were less than 0.1 IU/mL. Therefore, the above results suggested that the detection of serum ASN concentration contributes to assess ASNase activity in ALL patients treated with ASNase.

## 4. Conclusion

A novel, rapid, and convenient LC-MS/MS method for analysis of ASN has been successfully developed and validated in the human serum. A one-step protein precipitation process using methanol was a convenient sample preparation method employed to prepare serum samples for LC-MS/MS analysis. The HILIC column allowed for excellent separation of the analytes, and symmetrical peak responses were obtained using a mobile phase of methanol-water (95 : 5, v/v) containing 0.1% formic acid and 5 mM ammonium formate. The total run time was 6 min, and no carryover was observed with an injection volume of 5 *μ*L. The method has been validated, demonstrating good specificity and sensitivity, appropriate recovery, and absence of matrix interference. The accuracy and precision over the concentration range of 2–200 *μ*M satisfied the guidelines. The robustness of the developed method was confirmed by monitoring ASN concentrations in serum samples from ALL patients. Therefore, the method employed herein is a simple, rapid, and selective analytical option, finding a potential utility for quantification of ASN in ALL patients treated with ASNase.

## Figures and Tables

**Table 1:** Matrix effect for the determination of ASN (*n* = 4).

Nominal conc. (*μ*M)	Matrix effect (%)	IS-normalized matrix effect
6	63.49 ± 4.94	100.91 ± 13.19
80	63.34 ± 1.16	94.64 ± 4.73
150	65.83 ± 0.53	96.10 ± 2.09

**Table 2:** Precision and accuracy for the determination of ASN (*n* = 6).

Nominal conc. (*μ*M)	Backcalculated conc. (*μ*M)	Accuracy (%)	Precision (CV, %)	
			Intrabatch	Interbatch
2	1.99 ± 0.27	99.44 ± 13.52	15.02	14.74
6	5.51 ± 0.45	91.80 ± 7.48	8.54	11.21
80	79.94 ± 4.15	99.93 ± 5.18	5.96	3.89
150	145.24 ± 6.83	96.82 ± 4.55	5.03	6.02

**Table 3:** Stability of ASN in precipitated human serum samples under different storage conditions (*n* = 3).

Storage condition	Recovery (%)	
	LQC (6 *μ*M)	HQC (150 *μ*M)
Benchtop stability (48 h)	101.89 ± 0.59	102.87 ± 0.98
Long-term stability (−70°C, 87 days)	96.83 ± 1.48	99.90 ± 9.22
Processed stability (autosampler, 48 h)	107.78 ± 11.05	101.54 ± 3.42
Processed stability (4°C, 48 h)	100.61 ± 4.02	97.39 ± 6.07
Benchtop stability (2 IU/mL ASNase, 48 h)	98.56 ± 0.54	93.76 ± 3.56

**Table 4:** ASN concentrations in serum samples of ALL patients treated with ASNase.

No.	ASN concentration (*μ*M)	
	Before treatment	After treatment
1	45.86	<2
2	39.52	<2
3	36.83	<2
4	69.72	<2
5	59.18	<2
6	45.52	<2
7	54.12	<2
8	66.14	<2
9	52.76	<2
10	46.51	<2
11	90.32	<2
12	44.22	<2
13	66.60	<2
14	64.34	<2
15	59.80	<2
16	41.95	<2
17	52.65	<2
18	51.04	<2
19	46.83	<2
20	38.37	<2
21	46.79	42.63
22	77.35	<2
23	42.41	<2
24	53.70	<2
25	36.16	<2
26	47.85	<2
27	60.55	<2
28	41.40	<2
29	43.47	<2
30	43.55	17.60
31	79.48	23.60
32	54.62	<2
33	38.25	<2
34	39.82	<2
35	38.39	<2
36	58.06	<2
37	NA	<2
38	NA	<2
39	NA	<2
40	NA	<2
41	NA	<2
42	NA	<2
43	NA	<2
44	NA	<2
45	NA	<2
46	NA	<2
47	NA	<2
48	NA	<2
49	NA	<2

NA: serum samples were not collected before administration.

## Data Availability

The data used to support the findings of this study are included within the article.
